# Characterisation of Titanium-Oxide Thin Films for Efficient pH Sensing in Low-Power Electrochemical Systems †

**DOI:** 10.3390/s25196113

**Published:** 2025-10-03

**Authors:** Zsombor Szomor, Lilia Bató, Orsolya Hakkel, Csaba Dücső, Zsófia Baji, Attila Sulyok, Erzsébet Dodony, Katalin Balázsi, János M. Bozorádi, Zoltán Szabó, Péter Fürjes

**Affiliations:** 1 Centre for Energy Research, Institute of Technical Physics and Materials Science, 1121 Budapest, Hungary; bato.lilia@ek.hun-ren.hu (L.B.); hakkel.orsolya@ek.hun-ren.hu (O.H.); ducso.csaba@ek.hun-ren.hu (C.D.); baji.zsofia@ek.hun-ren.hu (Z.B.); sulyok.attila@ek.hun-ren.hu (A.S.); dodony.erzsebet@ek.hun-ren.hu (E.D.); balazsi.katalin@ek.hun-ren.hu (K.B.); bozoradi.janos@ek.hun-ren.hu (J.M.B.); szabo.zoltan@ek.hun-ren.hu (Z.S.); furjes.peter@ek.hun-ren.hu (P.F.); 2 Doctoral School on Materials Sciences and Technologies, Óbudai University, 1034 Budapest, Hungary

**Keywords:** pH-sensing, atomic layer deposition, metal oxide, titanium-oxide, low power electronics

## Abstract

A compact electrochemical sensor module for pH detection was developed for potential integration into specialized devices used for live cell or tissue incubation, for applications in highly parallelized cell culture analysis, by incorporating Organ-on-Chip devices. This research focuses on the deposition, structural and chemical analysis, and functional characterization of different titanium-oxide layers with various compositions as potentially sensitive materials for pH sensing applications. The titanium-oxide layers were deposited using vacuum sputtering and atomic layer deposition at 100 °C and 300 °C, respectively. Transmission electron microscopy and X-ray photoelectron spectroscopy were utilized to determine the specific composition and structure of different titanium-oxide layers. These TiO_*x*_-functionalized electrodes were connected to the application-specific analog front-end chip of the low-power readout circuit for precise evaluation. The pH sensitivity of the differently modified electrodes, employing various TiO_*x*_ materials, was evaluated using pH calibration solutions ranging from pH 6 to 8. Among the various deposition solutions, such as sputtering or high-temperature atomic layer deposition, the TiO_*x*_ layer deposited using low-temperature atomic layer deposition proved more suitable for pH sensing applications, with a sensitivity of 54.8–56.7 mV/pH, which closely approximates the Nernstian response.

## 1. Introduction

In addition to various biomarkers, such as relevant amino acids, carbohydrates, and antibiotics, pH stands out as a key factor for characterizing the metabolic conditions of cell cultures and tissues in Organ-on-Chip systems [1,2]. The applicability of the optical analysis method for measuring pH in a Phenol Red dye-containing medium was proved; however, an electrochemical method implementing an architecture that is integrable and applicable for various culture media could be beneficial due to its linearity in pH compared to other optical methods [3].

Miniaturized and robust pH sensors have been reported that use potentiometric and conductometric methods as well as transistor-based configurations, including the ion-sensitive field-effect transistor (ISFET) and the extended-gate field-effect transistor (EGFET). These devices make use of a wide range of ion-sensitive metal oxides (MO_x_), such as RuO_2_, IrO_2_, TiO_2_, SnO_2_, Ta_2_O_5_, WO_3_, V_2_O_5_, and ZnO [4,5,6,7,8,9,10]. ISFETs, in particular, represent the earliest and most established platform for pH detection [11,12,13,14,15]. They operate by translating changes in ionic activity at the sensing electrode–electrolyte interface into variations of the transistor current. Since their introduction, ISFETs have gained prominence for both chemical and biological analysis [16] and, beyond pH, have also been employed to monitor ion concentrations, such as sodium (Na^+^) and potassium (K^+^), in solution [17].

Despite these advantages, ISFET devices face critical drawbacks, including time-dependent instability, difficulties with encapsulation, and limited robustness for long-term use. To mitigate these problems, the EGFET configuration was proposed [18,19,20,21,22]. In this design, the chemically sensitive surface is decoupled from the MOSFET channel and externally connected, thereby protecting the transistor gate from direct exposure to the measurement environment. This arrangement improves stability, allows for the flexible integration of sensing layers, and enables the development of miniaturized and portable devices. Nonetheless, the EGFET still relies on an external reference electrode, which is often bulky, fragile, and costly, posing challenges for on-site and compact applications.

A key factor governing the response of EGFET-based pH sensors is the electrochemical behavior of their oxide sensing layer. The pH dependence arises from protonation/deprotonation reactions and the specific adsorption of H^+^/OH^−^ ions at the oxide–electrolyte interface, which modulate the surface charge and, consequently, the device output. Among the available MO_x_ candidates, TiO_2_ is one of the most extensively studied and reliable for this purpose. Different fabrication strategies have been employed to prepare such oxide layers, including sol–gel synthesis [23], electrodeposition [24], screen-printing [25], vacuum sputtering [26], and, more recently, inkjet printing [27]. Their sensing properties are commonly characterized using potentiometric measurements, cyclic voltammetry, and electrochemical impedance spectroscopy [28].

Titanium dioxide (TiO_2_) is a versatile material with many applications due to its appealing properties [29,30,31]. It is biocompatible, non-toxic, has efficient electronic charge properties, excellent adherence to the substrate’s surface, and can be functionalized with different biomolecules that make it an ideal candidate for biosensing applications. Nanostructured TiO_2_, appearing in the form of structured thin films, nanowires, or nanorods, has enhanced sensing characteristics owing to its higher surface-to-volume ratio [32,33,34,35]. Its sensing characteristics are predominantly ruled by the defects present in the amorphous or crystalline phases, which are determined by the applied deposition and annealing methods [36]. These layers are prepared using anodization, sol-gel method, hydrothermal method, atomic layer deposition, or sputtering. Atomic layer deposition offers excellent control over elemental composition, doping, and crystallinity at low deposition temperatures, which enables the creation of non-stoichiometric TiO_*x*_ layers that have potential applications in pH sensing.

In this work, TiO_*x*_ thin films were fabricated using vacuum sputtering and atomic layer deposition (ALD) techniques at 100°C and 300°C to develop a potentially sensitive material for low-power pH sensors. Titanium oxide can exist in different stoichiometries, with TiO_2_ being the most stable and widely used form. However, deposition methods like ALD and sputtering can yield sub-stoichiometric TiO_*x*_ (x < 2), which contains oxygen vacancies that influence electrical conductivity and sensing performance.

These oxygen vacancies introduce localized electronic states within the bandgap of TiO_2_, which act as active sites for proton adsorption. Water molecules readily adsorb to these vacancies, forming hydroxyl groups that can interact with protons, thereby enhancing proton conduction and pH sensitivity. Furthermore, oxygen vacancies facilitate proton conduction by creating pathways for proton migration, which is essential for the operation of pH sensors [37,38].

In this study, the oxide stoichiometry was intentionally varied, and techniques such as XPS were used to confirm the film composition. The pH-sensing mechanism is governed by surface reactions involving H^+^ and OH^−^ ions, which are strongly influenced by the oxide’s stoichiometry and defect structure. The sensing layers were tested using a readout circuit based on the operation of EGFET pH sensors. These results may contribute to the development of efficient, low-power pH sensors for biomedical and environmental applications.

## 2. Experimental Section

### 2.1. Layer Deposition Methods

**Figure 1:**
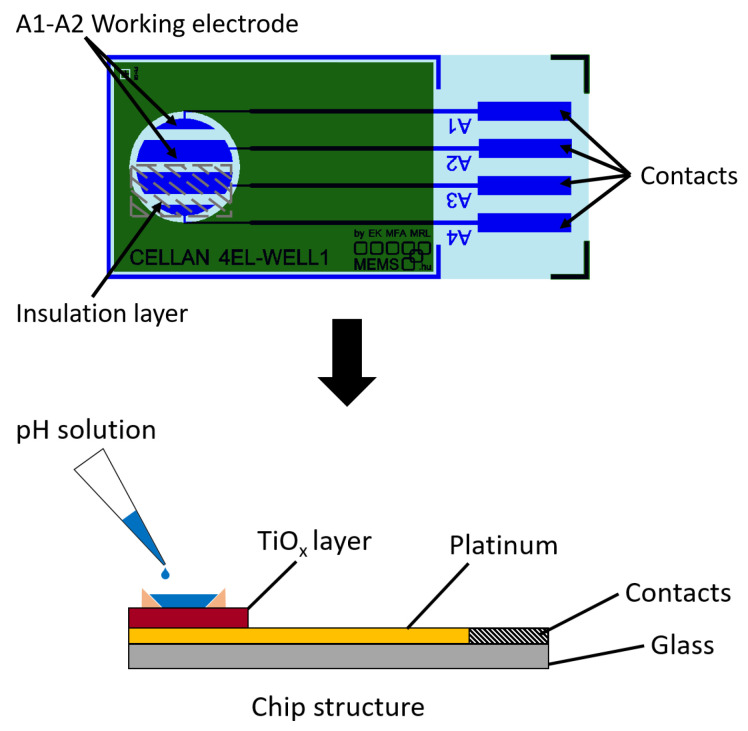
The configuration of the electrode with the deposited thin film. Borofloat glass serves as the substrate for the Pt electrodes, which were masked in areas linked to the A3 and A4 contacts, ensuring the TiO_*x*_ layer was deposited exclusively on the regions associated with the A1 and A2 contacts as working electrodes.

Electrochemical electrodes serve as the core components of various sensing and analytical devices. The integrated platinum (Pt) reference electrodes are crucial for maintaining a stable reference potential during electrochemical measurements. In contrast, the metal-oxide working electrodes are responsible for sensing and signal transduction. The device fabrication comprised the following steps: A borofloat glass substrate served as a stable and chemically inert foundation for electrode fabrication. Before deposition, it was treated with 65% HNO_3_ at 90 °C and rinsed with 18 MΩcm of deionized water. A 10 nm Ti adhesion layer was first applied, followed by a 160 nm Pt layer, which was sputtered and patterned using the lift-off technique with a 1.8 µm thick sacrificial photoresist to form the Pt electrodes. Next, the TiO_*x*_ layer was deposited using sputtering or atomic layer deposition (ALD) at 100 °C and patterned using the photoresist-based lift-off process to create a pH-sensitive layer on selected Pt electrodes. In the case of ALD deposition at 300 °C, the sacrificial layer was aluminum. The sputtered TiO_2_ layers were created via reactive sputtering in a 1 Pa vacuum, using an Ar:O_2_ gas mixture with a 1:1 ratio. The ALD metal-oxide layers were synthesized in a Picosun Sunale R-100 ALD reactor, utilizing titanium tetraisopropoxide (Sigma-Aldrich, Darmstadt, Germany), with water (>18 MΩcm) as the oxygen source. A 5 N purity nitrogen gas (supplied by Messer Inc., Budapest, Hungary) served as both the carrier and purging gas. The electrode configuration and the structure of the deposited layers are shown in Figure 1.

### 2.2. Characterization of TiO_x_ Layers

A variety of sophisticated electron microscopy and spectroscopy techniques were utilized to comprehensively analyze the deposited TiO_*x*_ layers. Transmission electron microscopy (TEM) investigations were carried out using an aberration-corrected Thermo Fisher Themis microscope (Philips CM 20, Graz, Austria) operated at 200 keV and equipped with a Super-X EDS detector (Graz, Austria). The cross-sectional TEM samples were prepared using the focused ion beam sample preparation technique. X-ray photoelectron spectroscopy (XPS) was employed to accurately ascertain the chemical composition and chemical states of the elements. XPS analysis was carried out using Escalab Xi+ (produced by Thermo Scientific, Massachusetts, USA) equipment. It operates at a base vacuum level of 2·10^−10^ mbar. Good resolved spectra were recorded at a 0.5 eV energy resolution using a monochromatized Al *K*α source focused onto an area with a 0.5 mm diameter.

### 2.3. Manufacturing the Readout Circuit for the Measurements

For precise measurements, an LMP91200 Chemical-Sensing device was used (manufactured by Analog Devices, Massachusetts, USA) in combination with custom-designed electronics. The LMP91200 device is an analog front-end (AFE) detector designed for low-power, analytical sensing applications, specifically for two-electrode sensors. It provides most of the necessary functions to detect changes based on a delta voltage at the sensor. The AFE operates over a voltage range of 1.8–5.5 V and is optimized for low-power applications. Depending on the configuration, the LMP91200 consumes 50 µA of current during measurements. Its extremely low input bias current makes it ideal for combining with pH sensors, minimizing pH probe degradation when the supply voltage is absent. Two additional guard pins support the high parasitic impedance wiring. The AD5940/41 front-end unit of the LMP91200 device contains a low-power, dual-output, string digital-to-analog converter (DAC) for setting the sensor bias voltage and low-frequency excitation. It also supports chronoamperometric and voltammetry electrochemical techniques and includes a trans-impedance amplifier (TIA) for low-bandwidth current measurements.

**Figure 2:**
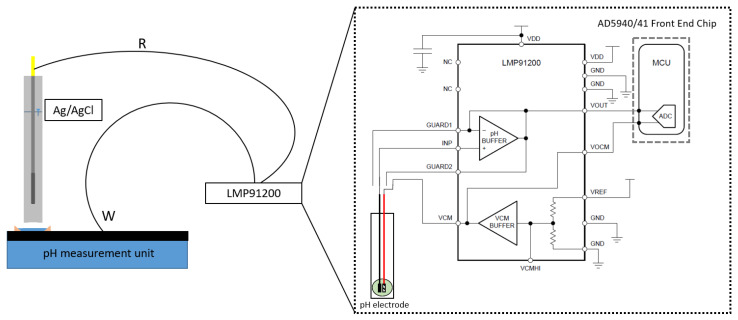
The measurement setup for the pH measurements using the LMP91200 Chemical-Sensing device with the AD5940/41 front-end chip.

During the measurements, the pH electrode was connected between the VCM pin and the INP pin. The voltage at the VCM pin represents the system’s internal zero, so the electrode potential (voltage at the INP pin) is referenced against the VCM voltage. Two guard pins (GUARD1, GUARD2) were provided to minimize input current leakage (Figure 2).

**Figure 3:**
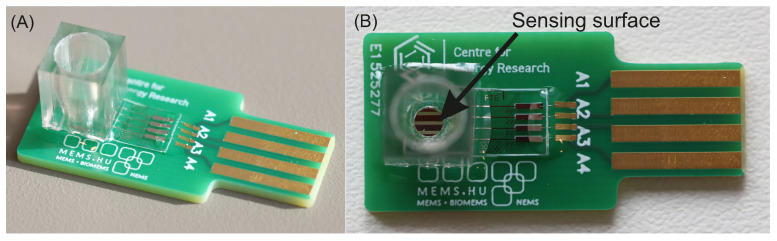
Perspective view of the electrochemical electrode platform showing the wire-bonded connections to the measurement unit (**A**). Top view of the device highlighting the sensing surface where the functionalized TiO_*x*_ thin films are located (**B**).

Once the electrode assembly was finalized, it was integrated into the sensor unit using wire-bonding technology (Figure 3) to provide reliable electrical connectivity, which is important for accurate signal acquisition and processing.

**Figure 4:**
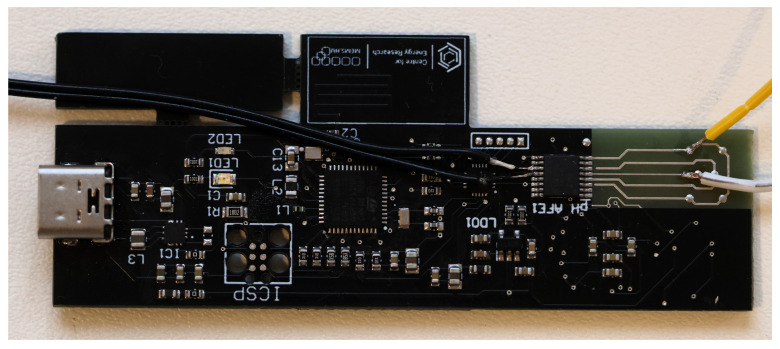
The low-power readout circuit with the AFE chip.

Attaching a polymer cuvette enhances usability by providing a stable and secure environment for holding liquid samples during electrochemical measurements. This attachment was achieved using a UV-curable adhesive to ensure durability and integrity, minimizing the risk of sample contamination or leakage, which is essential for maintaining experimental accuracy and reproducibility. The sensing unit with the electrochemical electrodes was combined with a specialized analog front-end chip (AFE) tailored for a low-power readout circuit (Figure 4). The AFE chip plays a crucial role by converting the analog signals produced by the electrodes into digital data, ensuring that the data are interpreted accurately and reliably. It is a sophisticated interface that optimizes signal processing capabilities, enabling efficient data acquisition and interpretation. This integration is essential for various applications, including environmental monitoring, biomedical diagnostics, and industrial process control, where accurate real-time measurements are paramount.

## 3. Results and Discussion

**Figure 5:**
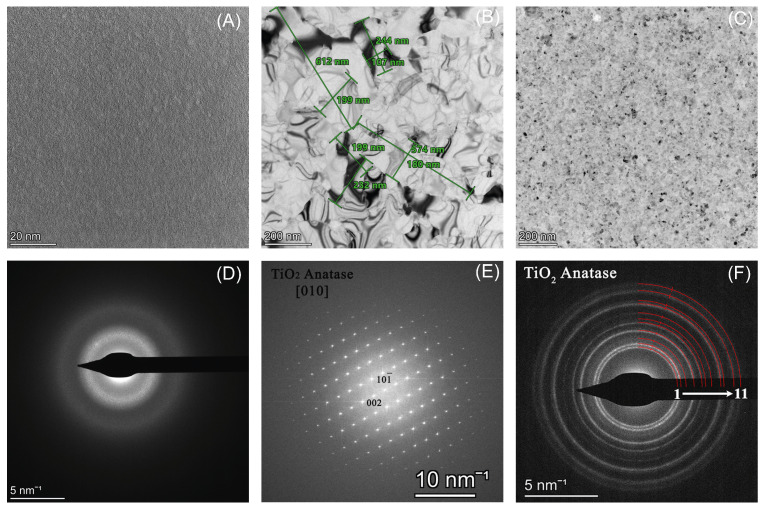
Bright−field TEM images of the different titanium-oxide layer surfaces deposited by ALD at 100 °C (**A**) and 300 °C (**B**) and by vacuum sputtering (**C**). The corresponding diffraction patterns in (**D**,**F**) and the Fourier transform of the HRTEM image in (**E**) served as a solid foundation for phase identification. The titanium-oxide layers deposited by ALD at 100 °C (**D**) exhibited an amorphous crystallized structure. In comparison, those deposited by ALD at 300 °C (**E**) and the sputtered layer (**F**) displayed a polycrystalline anatase structure with a grain size of a few hundred nanometers for the ALD and a few tens of nanometers for the sputtered layer. In image F, polycrystalline diffraction rings 1−11 correspond to the following hkl peaks of anatase in order: 011, 003, 012, 004, 112, 113 and 014, 020,121, 023, 024, 116.

As a result of TEM measurements, bright-field images revealed the differences in grain size among the three samples. The fast Fourier transforms of the high-resolution images (HRTEM) and the selected electron diffraction patterns served as a basis for determining the phase and structure. XPS measurements were also used to determine the Ti:O ratio in each case. The ALD TiO_*x*_ layers were all smooth and uniform on a 4” wafer. The growth rate was 0.3 Å/cycle at both deposition temperatures. The TEM micrographs (Figure 5A–C), however, reveal a difference between the layer structures. The layer deposited by ALD at 100 °C is amorphous, while the other two are both polycrystalline anatase according to the BF images, Fourier transforms, and the diffraction patterns shown in Figure 5. The layer prepared by ALD at 300 °C, however, has a larger grain size compared to the sputtered sample.

**Figure 6:**
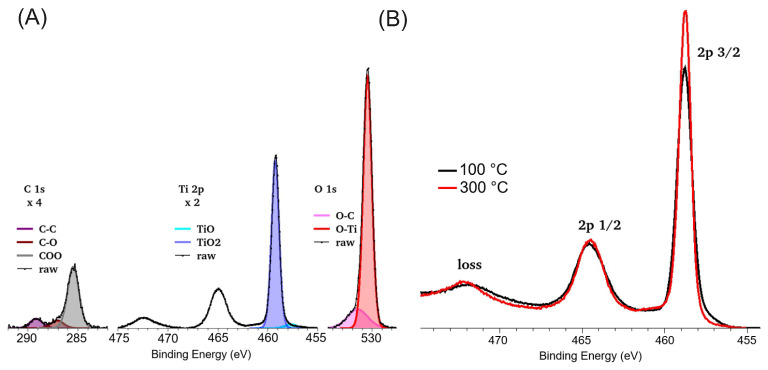
The XPS spectrum of the detected peaks corresponding to carbon, oxygen, and titanium atoms in the layer deposited by ALD at 100 °C (**A**). The components of different chemical states are presented with different color codes. On the right (**B**), Ti 2p peaks detected on both 100 °C and 300 °C layers are compared. The visible contribution at 457 eV comes from Ti^2+^, which is higher in the 100 °C layer. The 0.4 eV shift of the plasmon peak as a result of electron structure change also shows the difference in the materials deposited at 100 °C and 300 °C.

In terms of the XPS measurements, the detected peaks were Ti 2p 1/2 (464 eV) and 2p 3/2 (458 eV), O 1s (532 eV), and C 1s (284 eV). The peak intensities from the measured spectra were determined by peak fitting using Shirley background subtraction. The peak fitting also made possible the decomposition of complex peak shapes for components. The concentration calculation was performed on the evaluated area of peaks using the ALTHERMO library built into the equipment. The spectrum observed on the 100 °C sample can be seen in Figure 6, showing a TiO_2_-like material. The peaks are decomposed into chemical subcomponents. The carbon, which is partly surface contamination and partly built into the layer during deposition, can be separated into three subcomponents: C atom with C-C bond (visible at 285.4 eV), C-O bond (at 286.8 eV), and O=C-O bonds (at 289.2 eV). All energy values are 0.8 eV higher than nominal values due to surface charging. The two components of the oxygen peak originate from TiO_2_ at 530.5 eV and from organic compounds with oxygen content at 531.5 eV. The Ti 2p 3/2 peak has a dominant peak at 459 eV from Ti^4+^ while there is a small contribution at 457.5 eV from Ti^2+^. This is the consequence of oxygen deficiency in this layer. It is expressed more in the layer deposited at 100 °C than in the one at 300 °C. This difference is more visible in Figure 6B, where the titanium peak region is shown for the two layers for comparison. The plasmon energy also differs for the two layers (100 °C: 12.7 eV; 300 °C:13.1 eV), and is visible as a shift of the plasmon loss peak at 472 eV. This is a qualitative marker of the alteration of the electron structure of the TiO_*x*_ matrix. The change in oxygen content has a quantitative record in the calculated composition. Excluding the carbon content and the oxygen that is bound to carbon, the titanium/oxygen concentration can be calculated as 64% and 68% oxygen for the 100 °C and 300 °C layers, respectively. Although the absolute values may have a larger (few %) scale error, the measurement itself was reliable with low variance (1%) of data regarding measurements carried out at several different positions on the deposited layer. Thus, we can conclude that the 100°C layer has significantly less oxygen content than the 300 °C layer.

The XPS elemental analysis revealed that the layers deposited by ALD at 300 °C and vacuum sputtering showed Ti:O atomic ratios of 1:2.16 and 1:2.03, respectively. On the contrary, the ALD layer prepared at 100 °C has the least stoichiometric composition with a Ti:O atomic ratio of 1:1.76, showing the deviation from the ideal stoichiometric ratio typically expected for titanium-oxide compounds. In other words, this layer has a deficiency of oxygen compared to the stoichiometric composition (TiO_2_). This non-stoichiometric nature can impact the properties of the material, such as its electrical conductivity, chemical reactivity, and overall stability. The deviance from the stoichiometry can be explained by the deposition condition since the 100 °C deposition temperature is slightly outside the ideal ALD window, where the chemical reactions are not fully completed.

**Figure 7:**
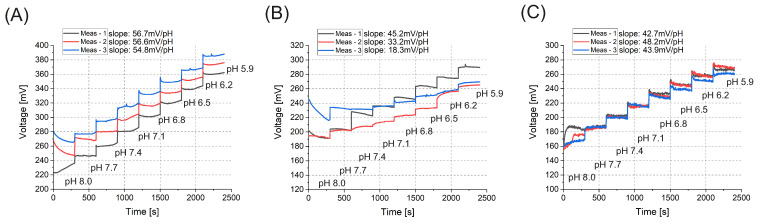
pH response of TiO_2_ layers fabricated using different deposition methods. (**A**) The ALD-deposited layer at 100 °C exhibited the highest pH sensitivity, showing a near-Nernstian slope of 59.1 mV/pH and excellent reproducibility across repeated measurements. (**B**) The ALD layer deposited at 300 °C showed moderate sensitivity with lower and more variable slopes. (**C**) The sputtered TiO_2_ film also demonstrated a moderate but more consistent pH response compared to (**B**). The voltage offsets were adjusted in the plots to visually distinguish individual measurement curves.

To assess the pH responsiveness of the sensor unit, a variety of titanium-oxide compositions were analyzed by testing with a series of pH calibration solutions in the range of pH 6–8 (Figure 7). For the measurements, a di-sodium hydrogen phosphate (Na_2_HPO_4_ · 12H_2_O) solution was combined with sulfuric acid via titration across the 6–8 pH range. Calibration was carried out using the Mettler Toledo Seven Compact pH meter, along with the InLab Expert Pro-ISM pH combi electrode and standard buffer solutions. Prior to each measurement, the sensing surface was rinsed with distilled water, and measurements were conducted at room temperature. At each layer, multiple measurements were taken to assess reproducibility to guarantee that the obtained pH values were consistent and repeatable. Factors such as sensor calibration, environmental conditions, and operator technique can influence reproducibility. Evaluating reproducibility helps to verify the reliability of the measurement process and to ensure that the pH sensor or the used method can consistently provide accurate results across multiple tests or experiments.

The output voltage vs. pH slope values in the case of layers deposited by ALD at 100 °C in the graph (Figure 7A) closely approximate the ideal Nernstian response of 59.12 mV/pH, suggesting that the layer exhibits a structure or surface characteristics that are optimized for efficient pH sensing. The highly linear and consistent response to pH variations shows enhanced sensitivity. The ALD process at 100 °C likely resulted in an amorphous TiO_*x*_ layer, providing improved uniformity and superior surface interactions. Lower deposition temperatures also minimize thermal stresses, which could otherwise lead to defects or irregularities in the film. For layers sputtered and ALD deposited at 300 °C (Figure 7B,C), the pH sensitivity is moderate, as shown by slope values remarkably deviating from the Nernstian response. This could be attributed to the polycrystalline nature of the layers, which may cause less uniform ion exchange or weaker interactions with H^+^= ions. Although higher temperatures can enhance crystallinity, they may also result in larger grain boundaries and irregular surface textures. These characteristics can diminish sensitivity and lead to variability across different areas of the electrode.

**Figure 8:**
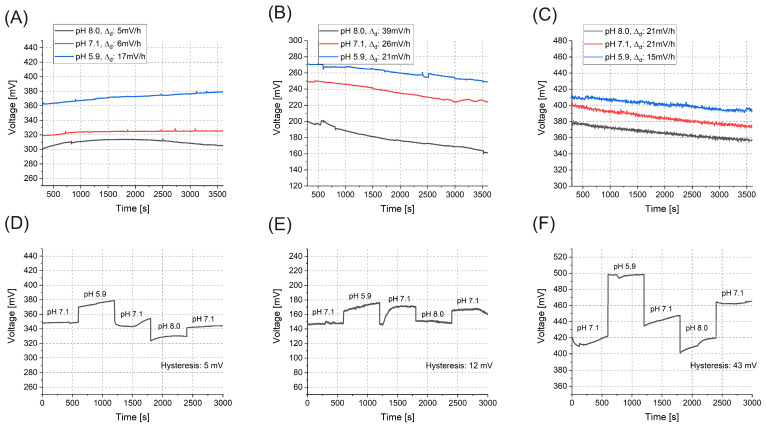
Drift and hysteresis measurements of TiO_*x*_ layers. (**A**–**C**) Drift behavior at fixed pH levels over 1 h for ALD 100 °C (**A**), ALD 300 °C (**B**), and sputtered (**C**) samples. The drift values ($\Delta_{d}$) represent the absolute change in voltage from the initial pH condition. The ALD 100 °C layer shows the lowest and most stable drift values. (**D**–**F**) Hysteresis evaluation by cycling pH from 7.1 to other values and back. The 100 °C ALD layer exhibits minimal hysteresis (5 mV), compared to 12 mV and 43 mV for the 300 °C and sputtered layers, respectively.

In addition to assessing pH sensitivity, measurements of drift and hysteresis were also conducted (Figure 8). Drift in pH sensors refers to the gradual change in the output signal of the sensor over time, even in the absence of any change in the pH of the solution being measured. It can be caused by factors such as changes in temperature, aging of sensor components, or variations in the reference electrode potential. The importance of hysteresis measurements for pH sensors refers to the difference in pH readings obtained when the pH of a solution is increased and then decreased back to its original value. It arises due to reversible chemical reactions or physical changes occurring at the electrode–solution interface, which may not immediately revert to the initial state upon reversing the pH change. Hysteresis measurement helps with understanding the sensor’s response characteristics and ensures that pH measurements are consistent and reproducible under varying pH conditions. Thus, by applying drift and hysteresis measurements alongside pH sensitivity evaluations, one can comprehensively assess the performance and reliability of pH sensors. This ensures that the sensors provide accurate and consistent pH measurements over time and under changing environmental conditions, making them suitable for various practical applications.

Drift measurements revealed that the layer deposited by ALD at 100 °C (Figure 8A) exhibited the greatest stability, with much less variation over time. On the contrary, the ALD layer deposited at 300 °C (Figure 8B) and the sputtered layers (Figure 8C) exhibited noticeably higher drift, indicating increased instability during the measurement process. This reduced stability in the latter methods may be attributed to structural imperfections or less favorable surface properties, which can result in inconsistent sensor responses under the same conditions. In a similar vein, the hysteresis measurements confirmed the superior performance of the ALD layer deposited at 100 °C (Figure 8D), which exhibited the smallest deviations when transitioning between pH 7 and other pH levels. This behavior suggests that the layer produced at 100 °C offers a more stable and reliable response to environmental changes. In contrast, the ALD layer deposited at 300 °C (Figure 8E) and the sputtered layers (Figure 8F) showed greater hysteresis, highlighting their limited ability to maintain consistent performance under fluctuating pH conditions.

The pH sensor resolution was estimated as the ratio of voltage uncertainty (due to noise, drift, and hysteresis) to pH sensitivity. With a readout resolution of ∼1 mV, the theoretical resolution of the near-Nernstian ALD layer at 100 °C is 1mV/(59mV/pH) ≈ 0.017 pH. Practically, hysteresis and drift dominate: the 100 °C ALD layer exhibited 54–56 mV/pH sensitivity, ∼5 mV hysteresis (∼0.04–0.05 pH), and drift of 0.1–0.3 pH/h, giving an effective short-term resolution of ∼0.05 pH. In comparison, the 300 °C ALD layer had lower, variable sensitivity (18–45 mV/pH), ∼12 mV hysteresis (0.3–0.4 pH), and 0.7–1.2 pH/h drift. The sputtered TiO_2_ film showed 43–48 mV/pH but large hysteresis (∼1 pH) and drift (0.3–0.5 pH/h), yielding substantially poorer resolution.

**Figure 9:**
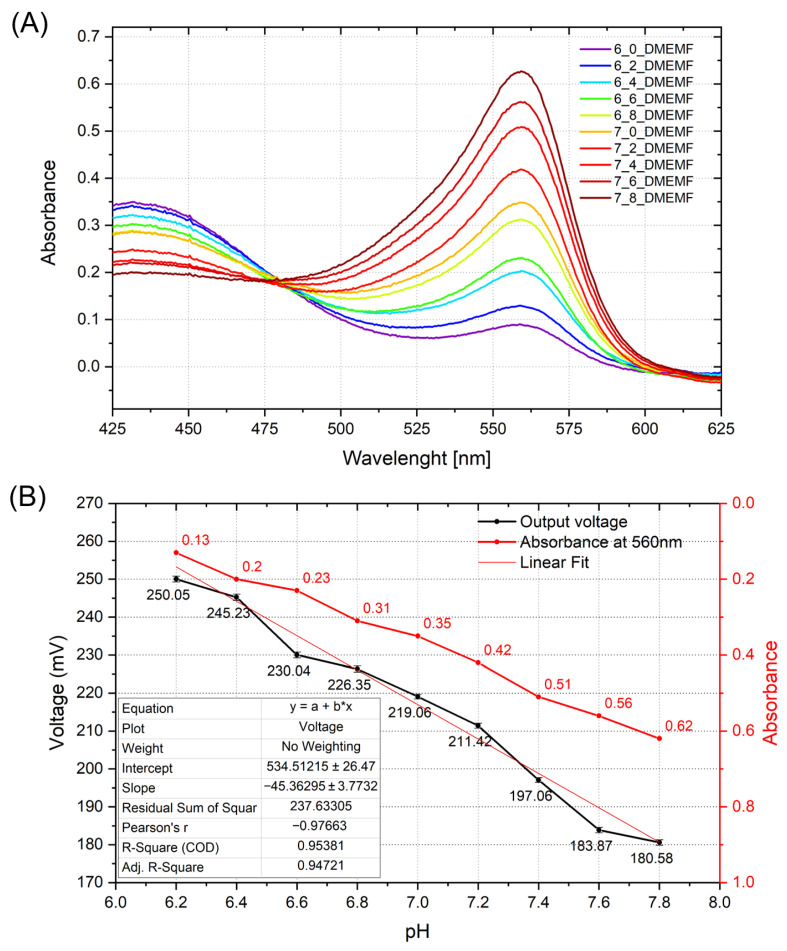
Absorbance spectrum (**A**) of the DMEMf solution and the corresponding output of the readout circuit with the absorbance values at 560 nm (**B**).

To evaluate the performance of the TiO_*x*_ layer deposited at 100 °C, an additional measurement was conducted to simulate biologically relevant pH fluctuations. This measurement replicated the accumulation of lactic acid in a cell culture medium during intense physical activity. The results are presented in Figure 9.

In this scenario, precise pH monitoring is essential for assessing cellular metabolism, fatigue, and acidosis. Lactic acid, a common byproduct of anaerobic respiration, lowers extracellular pH, making it a suitable agent for testing pH responsiveness [39,40,41]. In this experiment, lactic acid was titrated into DMEMf (Dulbecco’s Modified Eagle Medium with phenol red), a culture medium widely used for mammalian cells. The pH of the medium was adjusted in 0.2-unit steps within the range of 6.0–8.0, and each pH value was confirmed using a previously applied Mettler Toledo Seven Compact pH meter. The output voltage of the sensor was measured under steady-state conditions at each step. Concurrently, optical absorbance measurements were taken using UV-Vis spectroscopy with an AvaSpec-2048 Fiber Optic spectrometer (Avantes, Apeldoorn, The Netherlands). The spectroscopy was recorded at a wavelength sensitive to biochemical changes in the medium. The absorbance spectrum (Figure 9A) reveals a clear and consistent increase in optical density, especially at around 560 nm as pH decreases, indicating the medium’s sensitivity to lactic acid-induced acidification. This trend validates the suitability of optical absorbance as a complementary method for monitoring pH changes in biologically relevant environments. Investigating the output of the readout circuit (Figure 9B), the output voltage (black curve) showed a strong linear relationship with decreasing pH (R^2^ ≈ 0.95) with a response of 45.36 mV/pH, confirming the response of the TiO_*x*_ layer to pH changes in the DMEMf solution. Similarly, optical absorbance at 560 nm (red curve) increased with the pH, reflecting the color change of the medium containing the fenol red indicator due to lactic acid buildup. This combined electrical and optical measurement confirms the sensitivity of the sensor to chemical changes and highlights its applicability as a complementary method to the optical measurements. The experiment demonstrates that the ALD-deposited TiO_*x*_ pH-sensitive layer can reliably detect pH shifts in a controlled and simulated, but physiologically relevant, environment.

## 4. Conclusions

The TiO_2_ layers deposited by sputtering and by ALD at 300°C exhibited moderate pH sensitivity, which can be attributed to their perfect stoichiometry and inert structure. In contrast, the TiO_*x*_ layer deposited by ALD at 100°C demonstrated exceptional suitability for pH sensing applications with a slope of 54.8–56.7 mV/pH, as it closely approximated the Nernstian response of 59.12 mV/pH (Figure 7A). The results of the reproducibility, sensitivity, drift, and hysteresis tests also highlight the superior performance of the TiO_*x*_ layer deposited at 100 °C. This layer demonstrated exceptional uniformity and stability and minimal fluctuations over time, ensuring consistent pH sensitivity and reliable performance. In contrast, the TiO_*x*_ layers deposited by ALD at 300 °C and using vacuum sputtering showed greater variation in slope values, higher drift, and increased hysteresis, likely due to structural inconsistencies and fewer optimal surface properties. These findings confirm that the low-temperature ALD process is the most suitable for creating stable and precise pH-sensing layers.

Surface analytical methods further verified that the selected titanium-oxide layer is non-stoichiometric, with a Ti:O atomic ratio of 1:1.76. This deviation from the stoichiometry with a Ti:O atomic ratio of 1:1.76 is a key factor in enhancing the pH sensitivity, as its oxygen vacancies or defects enhance the material’s ability to interact with protons, thereby leading to improved pH sensitivity. These results provide essential insights into the sensitivity and accuracy of TiO_*x*_-functionalized electrodes, highlighting their significant potential for various scientific applications where precise pH measurement is critically important. This research underscores the capability of these electrodes to deliver reliable and consistent performance, making them valuable tools in settings that demand high levels of accuracy and responsiveness.

## Data Availability

The data are contained within the article.

## Abbreviations

The following abbreviations are used in this manuscript:

ISFET	Ion Selective Field Effect Transistor
EGFET	Extended Gate Field Effect Transistor
MOSFET	Metal Oxide Semiconductor FET
ALD	Atomic Layer Deposition
TEM	Transmission Electron Microscopy
HRTEM	High Resolution Transmission Electron Microscopy
XPS	X-ray Photoelectron Spectroscopy
AFE	Analog Front End
DAC	Digital Analog Converter
TIA	Trans Impedance Amplifier
DMEM	Dulbecco’s Modified Eagle Medium
